# Copine A, a calcium-dependent membrane-binding protein, transiently localizes to the plasma membrane and intracellular vacuoles in *Dictyostelium*

**DOI:** 10.1186/1471-2121-6-46

**Published:** 2005-12-12

**Authors:** Cynthia K Damer, Marina Bayeva, Emily S Hahn, Javier Rivera, Catherine I Socec

**Affiliations:** 1 Biology Department, Vassar College, 124 Raymond Ave., Poughkeepsie, NY 12604

## Abstract

**Background:**

Copines are soluble, calcium-dependent membrane binding proteins found in a variety of organisms. Copines are characterized as having two C2 domains at the N-terminal region followed by an "A domain" at the C-terminal region. The "A domain" is similar in sequence to the von Willebrand A (VWA) domain found in integrins. The presence of C2 domains suggests that copines may be involved in cell signaling and/or membrane trafficking pathways.

**Results:**

We have identified six copines genes in the *Dictyostelium discoideum *genome, *cpnA-cpnF*, and have focused our studies on *cpnA*. CpnA is expressed throughout development and was shown to be capable of binding to membranes in a calcium-dependent manner *in vitro*. A GFP-tagged CpnA was also capable of binding to membranes in a calcium-dependent manner *in vitro*. In live wildtype *Dictyostelium *cells expressing GFP-CpnA, GFP-CpnA was typically found in the cytoplasm without any specific localization to membranes. However, in a small subset of starved cells, GFP-CpnA was observed to bind transiently (typically ~1–10 s) to the plasma membrane and intracellular vacuoles. In some cells, the transient membrane localization of GFP-CpnA was observed to occur multiple times in an oscillatory manner over several minutes. In plasma membrane disrupted cells, GFP-CpnA was observed to associate with the plasma membrane and intracellular vacuoles in a calcium-dependent manner. In fixed cells, GFP-CpnA labeled the plasma membrane and intracellular vacuoles, including contractile vacuoles, organelles of the endolysosomal pathway, and phagosomes.

**Conclusion:**

Our results show that *Dictyostelium *has multiple copine homologs and provides an excellent system in which to study copine function. The localization of a GFP-tagged CpnA to the plasma membrane, contractile vacuoles, organelles of the endolysosomal pathway, and phagosomes suggests that CpnA may have a role in the function of these organelles or the trafficking to and from them. In addition, we hypothesize that the observed transient oscillatory membrane localization of GFP-CpnA in a small subset of starved cells is caused by fast calcium waves and speculate that CpnA may have a role in development, particularly in the differentiation of stalk cells.

## Background

Copines are highly conserved, calcium-dependent membrane binding proteins found in a variety of eukaryotic organisms. Multiple copine homologs exist in each of *Paramecium*, *Arabidopsis*, *C. elegans*, mice, and humans. Copines are characterized as having two C2 domains at the N-terminal region followed by an "A domain" at the C-terminal region. The "A domain" is similar in sequence to the von Willebrand A (VWA) domain found in integrins. Following the A domain, copines have a variable length C-terminal domain, which may confer unique characteristics to the different copine family members [1].

The C2 domain is a calcium-dependent phospholipid-binding motif originally identified in protein kinase C. Single and multiple copies of C2 domains are found in a large number of eukaryotic proteins. Most proteins containing a single C2 domain are involved in signaling pathways; examples include protein kinases, lipid kinases, phospholipases, and GTPase activating proteins. In contrast, most proteins that have multiple C2 domains are involved in membrane trafficking. Some examples of multiple C2 domain proteins are synaptotagmin, rabphilin, DOC2, each of which have two C2 domains, and munc13, which has three C2 domains [2,3]. The VWA domain is named from the von Willebrand Factor, a plasma and extracellular matrix protein. VWA domains have been studied in integrins and several extracellular matrix proteins and appear to function as protein-binding domains [4]. Copines were the first intracellular proteins to be identified as having a VWA domain [1]. However, a recent sequence database search for VWA domains revealed that VWA domains are found in several other intracellular proteins present in all eukaryotes [4].

Copines possess several characteristics that suggest they may have a role in membrane trafficking. As described above, copines have two C2 domains, similar to other membrane trafficking proteins. Biochemical studies have shown that copines, like other C2 domain containing proteins, bind to phospholipids in a calcium-dependent manner [1,5-7]. In addition, the protein chromobindin 17, which binds to the secretory granules of chromaffin cells in the presence of calcium, has been identified as a copine [1]. However, no functional data exists to indicate a role for copines in membrane trafficking.

Although copines possess two C2 domains, studies with human copines indicate that copines are involved in calcium-dependent signaling pathways and are therefore, more functionally related to the single C2 domain proteins [8,9]. Using the A domain of several human copine proteins as bait in a yeast two-hybrid screening of a mouse embryo cDNA library, Tomsig et al. [8] isolated a wide variety of proteins, several of which are components of intracellular signaling pathways. Many of these interactions between the copine A domains and their target proteins were verified in *in vitro *pull-down assays. The authors hypothesized that copines may act to localize target proteins to a particular membrane in response to calcium. To test this idea, they used an *in vitro *assay to show that full-length copines were able to recruit target proteins to membranes in a calcium-dependent manner. In an *in vivo *assay, Tomsig et al. [9] used a dominant negative mutant copine construct consisting of only the A domain to inhibit signaling from the TNF-α receptor in human embryonic kidney 293 cells.

Copine mutants have been isolated in both *Arabidopsis *[7,10,11] and *C. elegans *[12]. *Arabidopsis *plants with loss of function alleles of one of the copines, *CPN1/BON1*, exhibit mutant phenotypes only under certain environmental conditions. Copine mutant plants are miniature at 22°C, but grow normally at 28°C. The miniature phenotype is due to a reduction in both the size and the number of cells in the plant [7]. In low humidity conditions, copine mutant plants are also smaller and display abnormal regulation of cell death, with small necrotic lesions on the leaves, an accelerated programmed cell death response, and increased resistance to pathogens [10].

In C. *elegans*, mutations in *nem-4*, which encodes a copine, are capable of suppressing loss of function alleles of *gon-2*. Suppression of *gon-2 *by *nem-4 *requires a low level of GON-2 activity. GON-2 is a cation channel required for postembryonic gonadal cell divisions and loss of function mutations in GON-2 lead to a sterile phenotype. The *nem-4 *mutant strains do not exhibit any obvious phenotype [12]. The data from these copine mutant studies in *Arabidopsis *and *C. elegans *suggest that copines may function in a wide variety of calcium-mediated signaling pathways that control processes such as cell growth, cell division, and cell death.

To further investigate the function of copines, we have chosen to study copines in the simple eukaryote *Dictyostelium discoideum *and have identified six copines genes in the *Dictyostelium *genome. *Dictyostelium *provides an ideal system for studying copine function for several reasons. First, although *Dictyostelium *lives as a single-celled amoeba, it contains multiple copine homologs and a comparison of each of the *Dictyostelium *copines with the other five indicates that they share only 28–60% identity in amino acid sequence. Therefore, the *Dictyostelium *copine genes are diverse in sequence and may carry out distinct functions. To study the function of each copine gene, single and multiple gene knockout mutants can be created by homologous recombination. Second, *Dictyostelium *are highly motile, phagocytic cells, possessing organelles and membrane trafficking pathways similar to mammalian cells. Therefore, *Dictyostelium *serves as a good model for studying membrane trafficking and a particularly good model for many of the phagocytic cells found in human tissues, in which copines are highly expressed [13,14]. Third, *Dictyostelium *executes a simple 24-hour developmental program to form multicellular fruiting bodies and thus, *Dictyostelium *provides a simplistic model to study copine function in programmed cell death and development.

Our studies have focused on *cpnA*, the most abundant copine gene cDNA in the *Dictyostelium *cDNA Sequencing Project database [15]. To characterize CpnA in *Dictyostelium*, we have determined the protein expression pattern of CpnA during development, examined the calcium-dependent membrane binding properties of CpnA, and expressed a GFP-tagged version of CpnA in wildtype *Dictyostelium *to determine the intracellular location of CpnA.

## Results

### Identification of six copine genes in *Dictyostelium*

**Figure 1:**
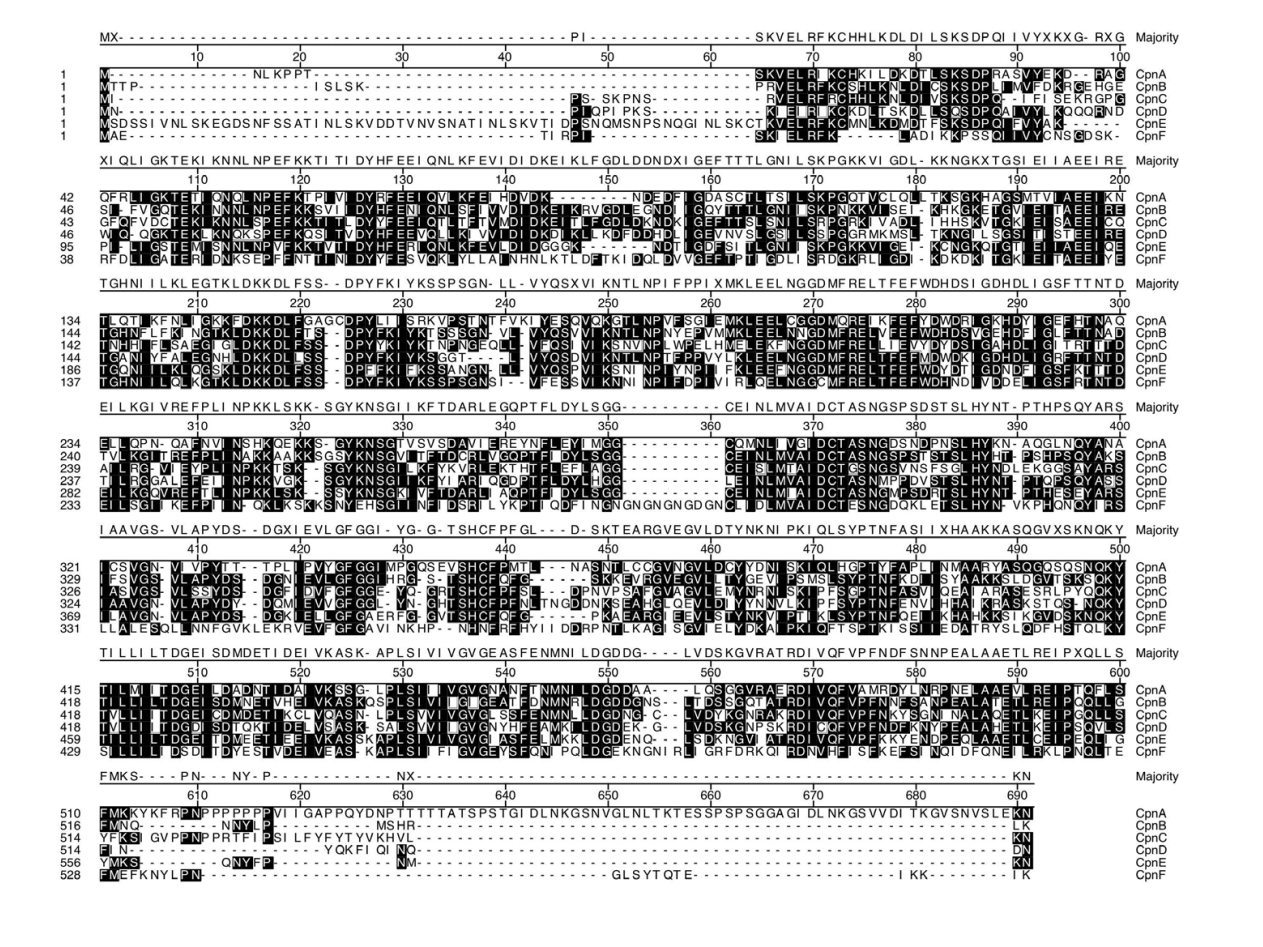
**Alignment of the amino acid sequences of the six copines genes in *Dictyostelium***. Amino acid sequences of CpnA and CpnB were derived from sequencing of full-length cDNA clones. The other four sequences were predicted from partial cDNA sequences and genomic sequences from the Japanese *Dictyostelium *cDNA Sequencing Project and *Dictyostelium *Genome Sequencing Project databases. A consensus sequence is shown above and residues matching the consensus are shaded.

To identify copine homologs in *Dictyostelium*, first we probed the cDNA sequence database from the *Dictyostelium *cDNA Sequencing Project at the University of Tsukaba in Japan [15] with the human copine I sequence. We initially found several different cDNA clones that exhibited some homology to human copine I. We obtained two full-length cDNA clones, sequenced them, and named the corresponding genes, *cpnA *[GenBank:AY332759] and *cpnB *[GenBank: AY5993970]. The open reading frame of the *cpnA *cDNA encodes a 600 amino acid protein, while the *cpnB *cDNA encodes a 530 amino acid protein. The complete cDNA sequences of *cpnA *and *cpnB *revealed two N-terminal C2 domains followed by an A domain characteristic of the copine family (Fig. 1). We then used the cDNA sequences of *cpnA *and *cpnB *to search both genomic and cDNA sequence databases for additional copine genes. We used these sequences to identify and predict amino acid sequences for four additional copine genes (*cpnC-F*) within the *Dictyostelium *genome. More recently, with the sequencing of the *Dictyostelium *genome complete, *cpnA*-*cpnE *have been identified by the *Dictyostelium *Sequencing Center gene predictions [16]. The alignment of the amino acid sequences derived from cDNA sequences of *cpnA *and *cpnB*, along with the predicted amino acid sequences of the four additional copine genes are shown in Fig. 1. All six copine genes contain the characteristic two C2 domains followed by an A domain and show 28–60% amino acid identity. Conserved sequences among the *Dictyostelium *copine proteins are found along the entire length of each protein (Fig. 1, shaded boxes). However, CpnA and CpnE contain unique additional sequences not found in each of the other five copine genes. CpnA has a longer C-terminal sequence, while CpnE has a longer N-terminal sequence (Fig. 1).

### Developmental expression pattern of CpnA

**Figure 2:**
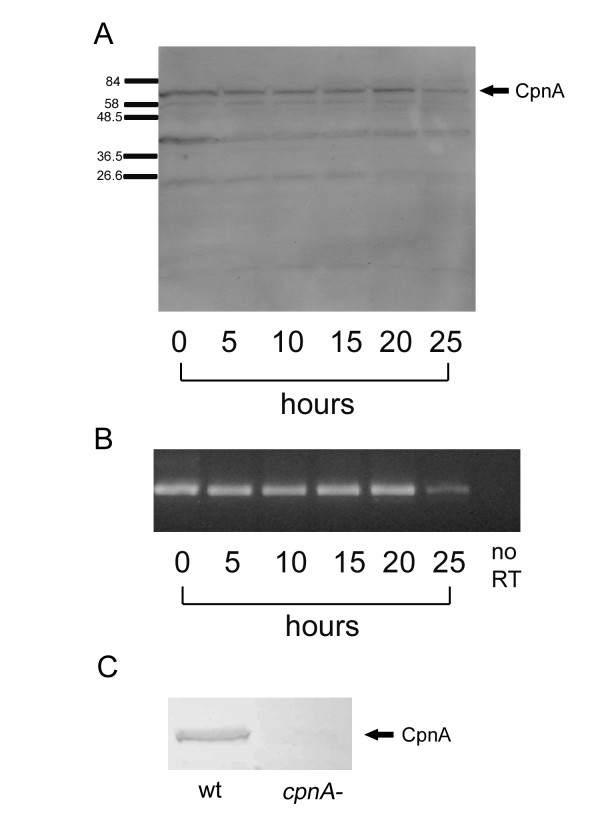
**CpnA is present throughout development**. Wildtype *Dictyostelium *cells in starvation buffer were placed on filters to develop. At 5-hour intervals, protein and RNA samples were harvested. A) Whole cell protein samples (5 × 10^6 ^cells per lane) from each time point were analyzed by Western blot using a purified CpnA antibody (arrow points to the 66 kDa CpnA). B) RNA samples from each time point were used in RT-PCR to detect *cpnA *mRNA. RT-PCR without the RT was run as a control (no RT). C) Whole cell protein samples (5 × 10^6 ^cells per lane) from wildtype cells and *cpnA*^- ^cells were analyzed by Western blot using the CpnA antisera (arrow points to the 66 kDa CpnA).

Because the *cpnA *gene was the most represented copine gene in the *Dictyostelium *cDNA Sequencing Project database, we have focused our work on CpnA. To determine if *cpnA *expression is developmentally regulated, we placed *Dictyostelium *cells of the NC4A2 strain, which we refer to as "wildtype," in starvation buffer and allowed them to develop on filters for 25 hours. At 5 hour intervals, we harvested protein and RNA samples and then used Western blotting to detect CpnA and RT-PCR to detect *cpnA *mRNA in these samples (Fig. 2). Western blots of whole cell samples using purified CpnA antibodies raised against a bacterially expressed fragment of CpnA recognized a protein band at the predicted molecular weight (65,877 Daltons) of CpnA, along with some smaller molecular weight protein bands (Fig. 2A). The 66 kDa CpnA band was observed in whole cell samples from each stage of development. Levels of CpnA are fairly steady throughout development, with slightly more CpnA found in vegetative cells (0 hr) and at the 20 hr time point when cells are beginning culmination and slightly less CpnA found at the 25 hr time point when cells have developed into mature fruiting bodies. The 66 kDa band recognized by the CpnA antiserum on Western blots using whole cell samples from wildtype cells was absent from whole cell samples made from *cpnA*^- ^null cells (Fig. 2C).

In addition to determining the presence of CpnA protein, we used RT-PCR to detect the presence of *cpnA *mRNA during development. We used RT-PCR to amplify a small region of *cpnA *across an intron to control for any contaminating DNA. mRNA from *cpnA *was amplified from each stage of development (Fig. 2B), suggesting that the *cpnA *transcript is present throughout development.

### CpnA binds membranes in a calcium-dependent manner *in vitro*

**Figure 3:**
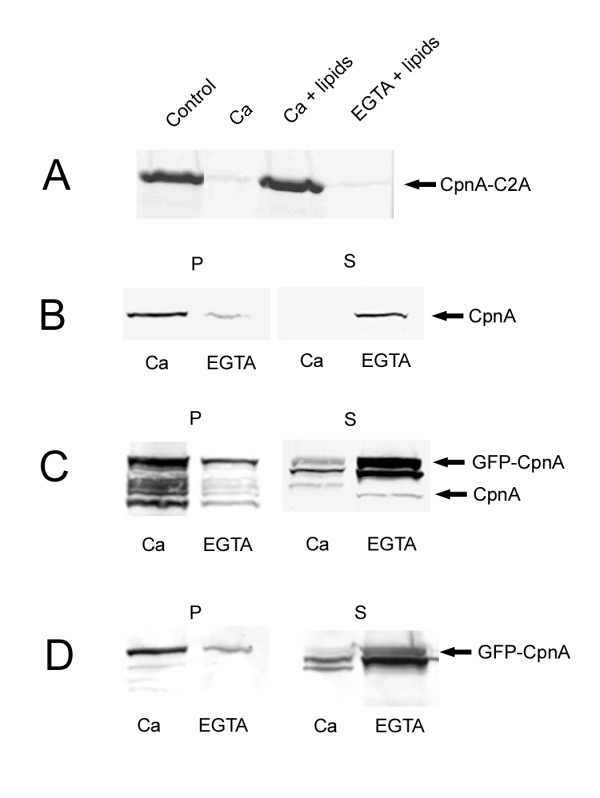
**CpnA binds membranes in a calcium-dependent manner**. A) A purified bacterially expressed fragment of CpnA containing only the first C2 domain (CpnA-C2A, 8 μg) was incubated with brain lipid liposomes in the presence of 2 mM calcium (Ca + lipids) or 2 mM EGTA (EGTA + lipids). The liposomes were centrifuged and liposomal pellets were analyzed on an 8% polyacrylamide-SDS gel stained with Coomassie Blue. The first two lanes are controls: 8 μg of CpnA-C2A was loaded on the gel (Control) in the first lane and protein pelleted in the presence of calcium, but absence of lipids (Ca) is in the second lane. B) Wildtype cells were disrupted with passage through a French Press and all membranes were pelleted in the presence of calcium or EGTA. Pellets (P) and supernatants (S) were analyzed by Western blot using CpnA antisera. C) Same procedure as in B) with cells expressing GFP-CpnA. The top arrow points to the GFP-CpnA band, while the bottom arrow points to the CpnA band. D) Same samples as in C) using a monoclonal antibody to GFP.

Because copines have two copies of the calcium-dependent phospholipid-binding C2 domain and *Arabidopsis *and mammalian copines have been shown to bind membranes in a calcium-dependent manner [1,5-7], we tested whether CpnA also possessed this ability. We began by trying to express the full length CpnA in bacteria as a GST-fusion protein; however, the bacteria did not express the fusion protein in any appreciable level. Therefore, we expressed and purified a fragment of CpnA containing only the first C2 domain. We used this fragment (CpnA-C2A) in *in vitro *phospholipid binding assays. The purified CpnA fragment was incubated with bovine brain lipid liposomes in the presence of calcium or EGTA and then centrifuged to pellet the liposomes. The pellets were analyzed by SDS-PAGE. CpnA-C2A pelleted with the liposomes in the presence of calcium, but was nearly absent in liposomes pelleted in EGTA (Fig. 3A), demonstrating that the first C2 domain of CpnA possesses the ability to bind lipids in a calcium-dependent manner.

Previous studies with synaptotagmin, a protein with two C2 domains, have shown that the lipid binding behavior of C2 domains is highly dependent on the type of phospholipids present and whether they are independent or paired with another C2 domain [17]. In addition, the two individual C2 domains of a neurally expressed mammalian copine have been shown to exhibit different calcium-dependent binding properties from each other [6]. Thus, it is important to investigate the membrane binding properties of the full length CpnA containing both C2 domains. Therefore, we examined whether endogenous CpnA binds to native *Dictyostelium *cellular membranes in a calcium-dependent manner. Wildtype *Dictyostelium *cells were disrupted with passage through a French Press, unbroken cells were pelleted, and then all membranes were pelleted by high-speed centrifugation in the presence of calcium or EGTA. The pellets and supernatants were then analyzed by Western blotting using the CpnA antisera. In the presence of calcium, CpnA was in the membrane pellet and completely absent from the supernatant (Fig. 3B). In the presence of EGTA, CpnA was mostly in the supernatant with a small fraction of the protein pelleting with membranes (Fig. 3B). These findings indicate that endogenous CpnA binds to native *Dictyostelium *membranes in a calcium-dependent manner.

### GFP-CpnA binds to membranes in a calcium-dependent manner *in vitro*

To determine the localization of CpnA within the cell, we expressed a GFP-tagged version of CpnA in wildtype *Dictyostelium *cells. GFP-CpnA is expressed under the *actin15 *promoter and is expressed at much higher levels than the native CpnA (see Fig. 3C, arrows). The overexpression of GFP-CpnA did not result in any easily identifiable phenotypes. Cells expressing GFP-CpnA grow normally in suspension and execute a normal developmental program (data not shown). The GFP was fused to the N-terminus to decrease the likelihood of the GFP interfering with the binding of target proteins to CpnA. However, because the C2 domains are in the N-terminal half of the protein, we tested whether GFP-CpnA has similar membrane binding properties to the endogenous CpnA. We performed the same membrane binding experiment as described above with cells expressing GFP-CpnA. GFP-CpnA behaved similarly to the native CpnA, with GFP-CpnA nearly absent from the supernatant in the presence of calcium (Fig. 3C). The small amount of GFP-CpnA found in the supernatant in the presence of calcium is most likely due to the overexpression of GFP-CpnA. Because we had problems with proteolysis of GFP-CpnA, we also analyzed the pellets and supernatants using a monoclonal antibody to GFP (Fig. 3D). These results indicate the addition of GFP to the N-terminus of CpnA does not disrupt the calcium-dependent membrane binding activity of CpnA (Fig. 3C, D).

### GFP-CpnA transiently localizes to the plasma membrane and intracellular vacuoles in a small subset of starved cells

In vegetative cells, GFP-CpnA was found throughout the cytoplasm without any specific localization to membranes. This was not surprising given that CpnA is a soluble cytoplasmic protein. It is known that cells that have been starved respond to cAMP with a transient rise in cytosolic calcium concentration [18,19]; therefore, we also imaged starved cells. Cells that have been starved for a several hours express cAMP receptors and use the synthesis and secretion of cAMP to signal their developmental program. After a few hours in starvation buffer, a few scattered cells will begin secreting periodic pulses of cAMP. Other cells undergo chemotaxis in response to the waves of extracellular cAMP, leading to the formation of aggregates of cells that differentiate into multicellular fruiting bodies consisting of a mass of encapsulated spores sitting atop a long thin stalk [20].

**Figure 4:**
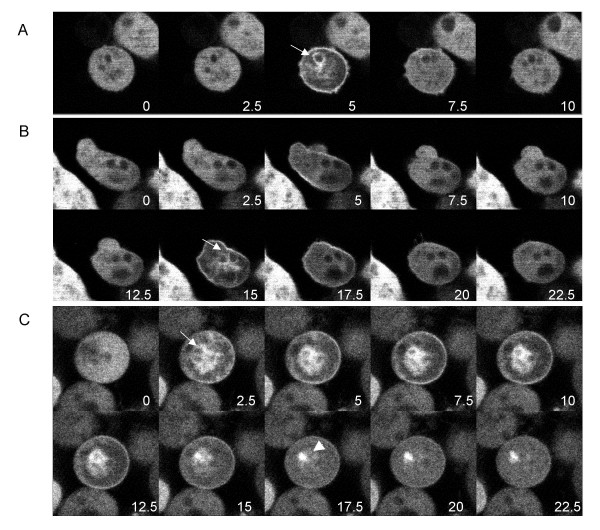
**GFP-CpnA transiently binds to the plasma membrane and intracellular vacuoles in a small subset of starved cells**. Cells expressing GFP-CpnA were washed three times in starvation buffer, placed on glass bottom plates, and imaged using a confocal microscope. A) and B) Successive time-lapse images taken every 2.5 seconds of a single cell from a plate of cells starved for 5.5 hours (arrows point to intracellular vacuoles). C) Successive time-lapse images taken every 2.5 seconds of a single cell from a plate of cells starved for 9.5 hours (arrowhead points to fluorescent dot). See Additional Files – Movies 1, 2, 3, 4, 5.

We placed cells expressing GFP-CpnA in starvation buffer on glass bottom dishes and imaged cells at various times after the initiation of starvation with a laser scanning confocal microscope. Scanning starved cells every 2.5 seconds, we observed that a small subset of cells beginning at 3–4 hours of starvation exhibited a very transient translocation of GFP-CpnA from the cytosol to the plasma membrane and then back to the cytosol (Fig. 4 and additional file 1). Cells that displayed this transient localization of GFP-CpnA to membranes were difficult to find; typically, only 1–4 cells in a field of 100 cells exhibited this behavior. However, cells exhibiting this behavior were found more frequently when the cells were starved for longer periods of time. In most cells, the membrane localization of GFP-CpnA was observed to last for only a few seconds before returning to the cytosol. This transient membrane localization of GFP-CpnA was frequently observed to occur multiple times in the same cell (Fig. 4B and additional files 2, 3, 4 and 5). It is not clear whether cells would continue to produce these oscillations of GFP-CpnA localization over a long period of time because we were only able to image cells for a few minutes before the cells became damaged by the laser. In most cells, we also observed localization to intracellular vacuolar structures (Fig. 4A, B, C, arrows). In addition, we often observed a centrally located fluorescent dot for a few seconds after the GFP-CpnA had returned to the cytoplasm (Figure 4C, arrowhead).

The cells that exhibited this transient GFP-CpnA membrane localization were often close to each other on the culture dish. After 9–10 hours of starvation, several cells within a small aggregate or adjacent to each other were seen to exhibit GFP-CpnA membrane localization at different times over several minutes (see additional file 4 for an example of adjacent cells each exhibiting this transient membrane localization of GFP-CpnA multiple times, yet, not synchronously). One cell exhibiting transient membrane localization of GFP-CpnA found within a small aggregate is shown in Fig. 4C (see additional file 5 for other cells within the same aggregate). In cells starved for 4–8 hours, membrane localization of GFP-CpnA occurred for ~1–10 seconds, while in cells starved for 8–10 hours, membrane localization sometimes occurred for longer periods of time ranging from ~1–30 seconds.

In an effort to determine if the transient localization of GFP-CpnA to membranes is triggered by cAMP being secreted by the starved cells, we tried globally treating starved cells with exogenous cAMP, which is known to produce a transient rise in intracellular calcium concentration. However, we did not observe a change in GFP-CpnA localization in starved cells when treated with cAMP.

### Membrane localization of GFP-CpnA is dependent on calcium during disruption of live cells

**Figure 5:**
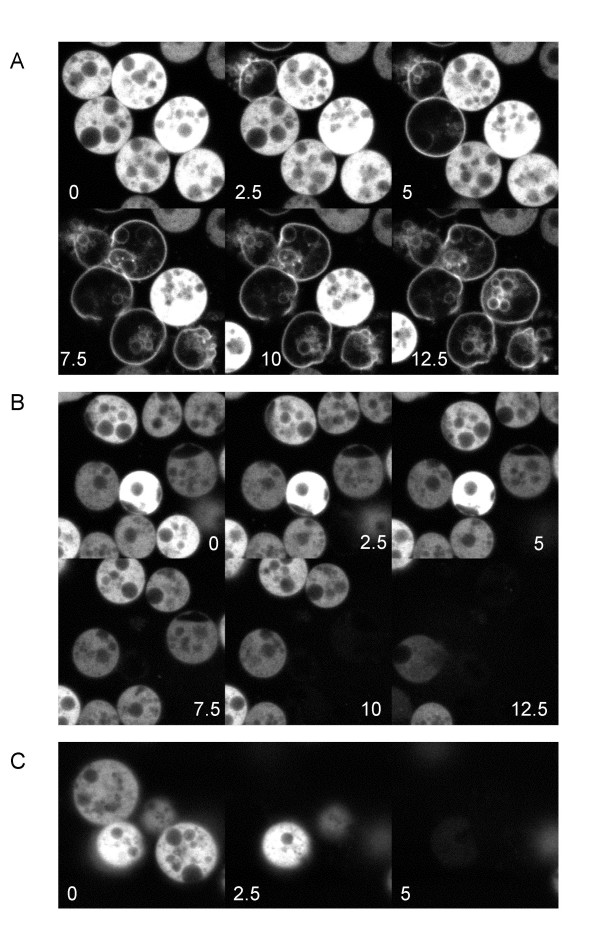
**GFP-CpnA associates with the plasma membrane and intracellular vacuoles in live disrupted cells in a calcium-dependent manner**. Cells expressing GFP-CpnA were placed in water (A) or water with 2 mM EGTA (B) and scanned every 2.5 seconds on a confocal microscope. C) Cells expressing GFP only were placed in water and scanned every 2.5 seconds on a confocal microscope. Images shown are time-lapse sequences of cells in which the plasma membrane has been disrupted. See Additional Files – Movies 6, 7, 8.

A stable membrane localization of GFP-CpnA was observed after prolonged exposure to the intense light of the laser of the confocal microscope in vegetative and starved cells. *Dictyostelium *cells are very sensitive to the heat produced by fluorescence microscopy. They lose their shape and adherence, becoming round and eventually bursting if exposed to the light for more than tens of seconds with a fluorescence microscope or frequent scanning over tens of minutes with a laser scanning confocal microscope. Using the light sensitivity of *Dictyostelium *to our advantage, we designed an assay to visualize GFP-CpnA in disrupted cells. First, we placed cells expressing GFP-CpnA or GFP alone in water, which resulted in an increase in the size of contractile vacuoles and an increase in the rate of plasma membrane disruption. We then scanned the cells every 2.5 seconds using confocal microscopy with 100% laser power (no attenuation). Over time the cells sustained plasma membrane ruptures, allowing soluble cytoplasmic proteins to diffuse out of the cell and into the water. In these broken cells, the GFP-CpnA was seen labeling the plasma membrane and intracellular vacuoles (Fig. 5A and additional file 6). In control cells expressing GFP alone, once the cell bursts, the GFP diffused into the water and the fluorescence disappeared with no labeling of membranes (Fig. 5C and additional file 8). To test whether the binding of GFP-CpnA to membranes in these broken cells is calcium-dependent, we repeated this experiment, but this time adding EGTA to the water. Once the cells burst, the GFP-CpnA did not bind to membranes, but diffused into the water and disappeared (Fig. 5B and additional file 7). GFP-CpnA binding to membranes was abolished by the EGTA indicating that the membrane binding activity of GFP-CpnA to the plasma membrane and intracellular vacuoles in these disrupted cells is calcium-dependent. In addition, binding of GFP-CpnA to the plasma membrane and intracellular vacuoles in these broken cells placed in water was similar whether or not additional calcium was added to the water. Because the calcium concentration of Millipore purified water is typically less than 5 μM, this suggests that GFP-CpnA's sensitivity to calcium is similar to mammalian copines, which have been shown to exhibit half maximal binding to membranes between 3 and 10 μM calcium *in vitro *[5].

### GFP-CpnA labels the plasma membrane and intracellular vacuoles in fixed cells

**Figure 6:**
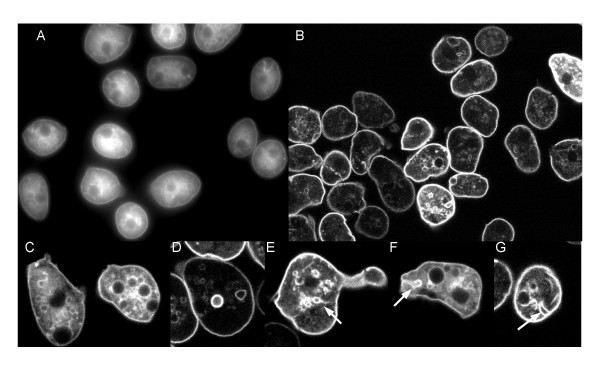
**GFP-CpnA associates with the plasma membrane and intracellular membranous vacuoles in fixed cells**. Cells expressing GFP-CpnA were flattened with agarose, fixed, and imaged using A) widefield fluorescence and B) confocal microscopy. Confocal images of single cells are shown in C, D, E, F, and G. Arrows point to labeled vacuoles with attachments to the plasma membrane. G) see Additional File – Movie 9 of z-series images.

In fixed cells, GFP-CpnA localized to the plasma membrane and intracellular membranous structures. Images of fixed cells look similar to images of disrupted cells and the subset of starved live cells that display the transient membrane localization of GFP-CpnA (Fig. 6). Thus, we used fixed cells to identify the GFP-CpnA labeled intracellular vacuolar structures. Intracellular structures labeled with GFP-CpnA in fixed cells included mainly rounded or vacuole type structures and less frequently tubular structures. The vacuolar structures were heterogeneous in size and found throughout the cytoplasm (Fig. 6A, B). The shape and size of some of these GFP-CpnA labeled structures is consistent with contractile vacuoles. The contractile vacuole system primarily functions in osmoregulation. Contractile vacuoles form a membranous network that consists of ducts, cisternae, and bladders. The bladders transiently form pores with the plasma membrane to expel water [21]. Using confocal microscopy, we found several cells that contained GFP-CpnA labeled structures with what looked like attachments to the plasma membrane (Fig. 6E, F, G, white arrows, see additional file 9 for z-series). These GFP-CpnA labeled structures forming attachments with the plasma membrane could be bladders caught in the act of expelling water.

### GFP-CpnA labels contractile vacuoles in fixed cells

**Figure 7:**
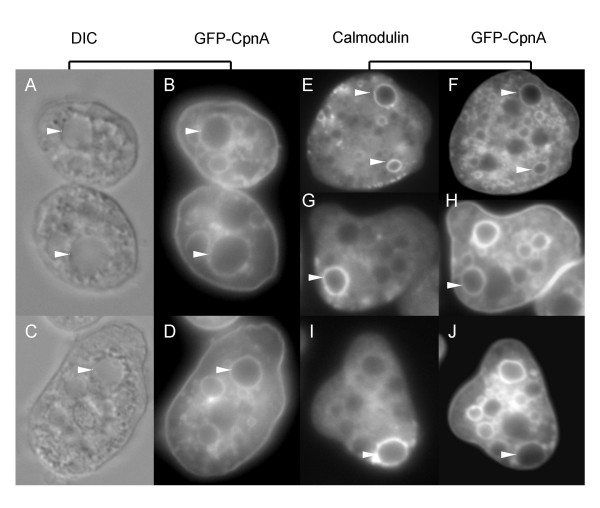
**GFP-CpnA labels contractile vacuoles**. Cells expressing GFP-CpnA were placed in water for 1.5 minutes, fixed, and imaged using differential interference contrast microscopy (A, C). These same cells were imaged for GFP with a widefield fluorescence microscope (B, D). Contractile vacuoles in cells expressing GFP-CpnA were labeled with a primary antibody to calmodulin and a TRITC-conjugated secondary antibody and then imaged for TRITC (E, G, I). These same cells were imaged for GFP (F, H, J). Arrowheads point to contractile vacuoles.

To definitively identify some of the GFP-CpnA labeled structures as contractile vacuoles, cells expressing GFP-CpnA were placed in water to cause an increase in the number and size of the contractile vacuoles. The cells were then fixed and imaged using differential interference contrast (DIC) and widefield fluorescence microscopy. Cells placed in water usually had one or two enlarged vacuoles that could be seen in the DIC images as smooth concave vacuoles (Fig. 7A, C). When imaged using fluorescence microscopy, these same vacuoles were outlined in GFP-CpnA (Fig. 7B, D). Calmodulin is a calcium-binding protein that is greatly enriched on contractile vacuole membranes in fixed *Dictyostelium *cells [22]. Using a monoclonal antibody to calmodulin, we performed immunofluorescence studies to label contractile vacuoles with rhodamine (TRITC) (Fig. 7E, G, I). The calmodulin antibody labeled contractile vacuoles corresponded exactly with some of the GFP-CpnA labeled structures (Fig. 7F, H, J). However, there were many more GFP-CpnA labeled structures than calmodulin antibody labeled structures, indicating that only some of the GFP-CpnA labeled structures are part of the contractile vacuole system. The numerous GFP-CpnA labeled structures not identified as contractile vacuoles are most likely part of the endolysosomal pathway, which includes endosomes, lysosomes, and postlysosomes. Postlysosomes are terminal organelles of the endocytic pathway that fuse with the plasma membrane to expel their contents [23]. The structures with attachments to the plasma membrane shown in Fig. 6 (E, F, G) could also be postlysosomes in the act of exocytosis.

### GFP-CpnA labels endolysosomal organelles and phagosomes in fixed cells

**Figure 8:**
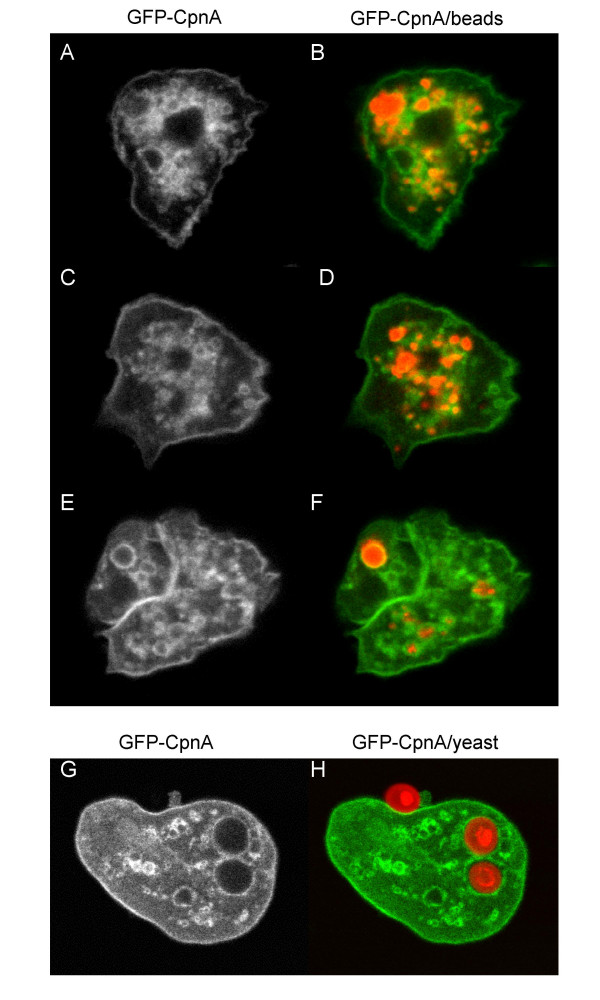
**GFP-CpnA labels endosomes, lysosomes, and phagosomes**. Cells expressing GFP-CpnA were incubated with red fluorescent nanobeads for 2 hours, flattened with agarose, fixed, and imaged for both the beads and GFP-CpnA simultaneously using confocal microscopy. The same cells are displayed twice; GFP-CpnA only is shown on the left (A, C, E), while both the red beads and GFP-CpnA are shown to the right (B, D, F). Cells expressing GFP-CpnA were incubated with Alexa Fluor-594-labeled yeast for 1 hour, flattened with agarose, fixed, and imaged for both the yeast and GFP-CpnA simultaneously using confocal microscopy. One cell is displayed twice: GFP-CpnA only (G) and to the right, both GFP-CpnA and yeast (H).

To identify some of the GFP-CpnA labeled structures as part of the endolysosomal system, we used red fluorescent nanobeads to label endosomes and lysosomes. Cells take up the beads by the processes of endocytosis and macropinocytosis. Endocytic vesicles form endosomes that become lysosomes, which eventually become postlysosomes [23]. Therefore, organelles all along the endocytic pathway are labeled with the red beads. Cells expressing GFP-CpnA treated with red nanobeads were fixed and imaged for both fluorophores simultaneously with confocal microscopy. To visualize the GFP-CpnA labeled structures more clearly, example images of GFP-CpnA expressing cells incubated with red nanobeads are displayed twice in Fig. 8. First, only the fluorescent signal from the GFP-CpnA is shown (Fig. 8A, C, E), and to the right, both the green and red signals are shown (Fig. 8B, D, F). In all cells examined, the red nanobeads corresponded exactly with GFP-CpnA, in the case of small vesicles, or were surrounded by an outline of GFP-CpnA, in the case of larger vacuoles. For the moderately sized vacuoles, it was sometimes necessary to change the plane of focus to image the GFP-CpnA labeled outline surrounding the beads. Not all GFP-CpnA labeled structures contained red beads; this is perhaps due to the fact that some of the structures are contractile vacuoles and that some are endosomes/lysosomes that do not contain beads. To get clear images of the GFP-CpnA labeled structures, it is necessary to flatten cells with a thin layer of agarose. However, flattening cells loaded with too many beads resulted in destroying the cells before fixation. Therefore, to view both GFP-CpnA and beads simultaneously, we had to use a bead concentration low enough so that cells remained intact during flattening, which resulted in many but not all organelles of the endolysosomal system containing beads.

Because it has been suggested that copines may be involved in phagocytosis [13], we also looked at whether GFP-CpnA localizes to the phagosome. Cells were fed Alexa Fluor-594-labeled yeast, fixed, and both fluorophores were imaged simultaneously using confocal microscopy. An example of one cell is shown in Fig. 8 (G, H). Again, for clarity, the cell is displayed twice; first only the fluorescent signal from the GFP-CpnA is shown (Fig. 8G), and to the right, both the green and red signals are shown (Fig. 8H). GFP-CpnA was found associated with the membrane of the phagosome, surrounding the phagocytosed yeast cell.

## Discussion

Copines make up a family of soluble, calcium-dependent membrane binding proteins found in a wide variety of eukaryotic organisms. Copines are characterized as having two C2 domains at the N-terminal region followed by an "A domain," similar to the von Willebrand A (VWA) domain found in integrins, in the C-terminal region [1]. Copines appear to be absent from the *Sacchromyces cerevisae *genome, while the genomes of *Paramecium*, *Arabidopsis*, *C. elegans*, and human have two, three, five, and nine, respectively [13,4,24]. Tomsig and Creutz [8] have hypothesized that copines play a role in calcium signaling by binding to target proteins with their A domains and then bringing those target proteins to a particular membrane through the action of their C2 domains in response to a rise in calcium concentration. Because copines have *two *C2 domains, they have also been hypothesized to have a role in membrane trafficking. It is possible that both ideas could be correct; copines may provide links between signaling pathways and membrane trafficking pathways.

Here, we have described the identification of six copine genes in *Dictyostelium *and the initial characterization of one copine gene, *cpnA*. CpnA is unique among the *Dictyostelium *copines in that it contains a C-terminal tail domain following the A domain that is ~50 amino acids longer (Fig. 1). Moreover, the C-terminal tail domain of CpnA does not exhibit sequence similarity to copines found in any organisms. Western blot analysis indicates that CpnA is present throughout the 24-hour development with only slight differences in protein levels (Fig. 2). However, any rapid changes in protein levels that may occur between the 5-hour time spans used in our experiment would not be detected. In *in vitro *binding assays, CpnA pellets with membranes in the presence of calcium. However, a small fraction of CpnA also pellets with membranes in the presence of EGTA, indicating that although most CpnA binds to membranes in a calcium-dependent manner, some may bind independently of calcium. This small amount of calcium-independent binding was also seen with the *Arabidopsis *copine protein [7] and a neurally expressed mouse copine, *N*-copine [6].

GFP-tagged copines have been expressed in both *Arabidopsis *[7] and *C. elegans *[12]. In *Arabidopsis*, CPN1-GFP localizes to the plasma membrane. In *C. elegans*, GEM-4::GFP also localizes to the plasma membrane; however, punctate staining of the cytoplasm is also observed. In vegetative *Dictyostelium *cells, a GFP-tagged version of CpnA was observed throughout the cytoplasm without any detectable association with membranes. This could perhaps be due to the fact that the GFP-CpnA is markedly overexpressed (see Fig. 3C) and the soluble GFP-CpnA filling the cytoplasm is masking any membrane association. However, we think this is unlikely, given that cells expressing GFP-CpnA display variable expression levels and imaging very low expressing cells did not reveal any membrane association. Alternatively, GFP-CpnA may only localize to membranes in response to a rise in the cytosolic calcium concentration.

When imaging a large field of starved cells, we noticed a few cells that displayed a very transient membrane localization of GFP-CpnA. In addition, the transient localization of GFP-CpnA often occurred multiple times within the same cell. Our observations that GFP-CpnA binds to membranes in a calcium-dependent manner *in vitro *and the rapid oscillatory nature of the translocation of GFP-CpnA from cytosol to membranes and back to the cytosol suggests that GFP-CpnA is responding to fast intracellular calcium spikes or waves. It is known that starved cells undergo oscillatory responses to waves of cAMP accompanied by a transient rise in calcium during the aggregation stage of development. However, these oscillations have a period of 6–7 minutes, while the GFP-CpnA membrane localization oscillations we have observed exhibit a period on the order of seconds or tens of seconds. On the other hand, fast oscillatory calcium spikes lasting for 10 to 30 seconds with a period of 1–2 minutes have been reported to occur specifically in regions containing prestalk cells in multicellular stages of *Dictyostelium *[25].

During differentiation, stalk cells vacuolate, synthesize a cellulose cell wall, and eventually undergo programmed cell death to form the thin stalk of the mature fruiting body [20]. Many lines of evidence indicate that calcium is involved in the differentiation of stalk cells [26]. Pre-stalk cells have been shown to possess higher levels of free calcium [25,27]. Moreover, vegetative cells that are newly starved fall into high and low sequestered calcium classes and those that possess higher levels of calcium tend to enter the prestalk regions of the slugs [28]. It has also been shown that increasing calcium levels increases the stalk cell to spore cell ratio [29,25]. The differentiation inducing factor, DIF, which induces the transcription of prestalk genes, has been shown to mediate its effects by causing a slow sustained increase in intracellular calcium levels [30].

We speculate that the observed oscillations of membrane localization of GFP-CpnA may be occurring in only pre-stalk cells that are accumulating higher levels of calcium. The higher concentrations of calcium in pre-stalk cells could produce oscillatory calcium spikes in an early pre-aggregative stage of development. If fast calcium waves are mediating the translocation of GFP-CpnA from cytosol to membranes as we have suggested, an important question that remains is what is triggering the calcium waves in these cells. The fact that global treatment of starved cells with cAMP does not induce GFP-CpnA membrane localization suggests that the trigger is not cAMP. The observed GFP-CpnA membrane oscillations could be triggered by other signaling molecules secreted by starved cells. Alternatively, the presumed calcium oscillations may be part of a stress response due to the strong illumination of the laser; some of the cells exhibiting these oscillations showed signs of blebbing caused by the laser.

In addition to transiently associating with the plasma membrane, GFP-CpnA was also seen to associate with intracellular vacuoles *in vivo*. A similar pattern was seen in fixed cells (Fig. 6); this may be caused by the release of calcium from intracellular stores during the fixation process. Using fixed cells, we were able to identify the GFP-CpnA labeled vacuoles as contractile vacuoles, organelles of the endolyosomal pathway, and phagosomes (Fig. 7 and Fig. 8). The association of GFP-CpnA with intracellular vacuoles suggest that CpnA may have a role in the function of these organelles and/or the trafficking between these organelles and the plasma membrane. Several multiple C2 domain-containing proteins have been implicated in membrane trafficking, particularly exocytosis [31]. In addition, it has been shown that in neutrophils fast calcium waves accompany phagocytosis [32].

## Conclusion

Our results indicate that *Dictyostelium *has multiple copine homologs and provides a good system in which to study copine function. CpnA is present in vegetative cells and throughout development suggesting it may function in all stages of development. *In vitro *assays show that CpnA is a calcium-dependent membrane binding protein. A GFP-tagged CpnA also binds to membranes in a calcium-dependent manner *in vitro*. GFP-CpnA translocates from the cytosol to specific membranes and back to the cytosol very rapidly in only a subset of starved cells *in vivo*. These membranes include the plasma membrane and intracellular vacuoles. Studies with fixed cells suggest these intracellular vacuoloes include contractile vacuoles, organelles of the endolysosomal pathway, and phagosomes. The association of GFP-CpnA with intracellular vacuoles suggest that CpnA may have a role in the function of these organelles. In addition, we speculate that the transient membrane localization of CpnA is caused by fast calcium spikes found only in cells destined to become stalk cells and hypothesize that CpnA may have a role in the differentiation of stalk cells. We have recently created a *cpnA*^- ^knockout mutant strain in *Dictyostelium *and characterization of the *cpnA*^-^mutants will allow us to test these hypotheses about the function of CpnA.

## Methods

### Strains and cell culture

The *Dictyostelium discoideum *strain used was NC4A2, an axenic strain derived from the wildtype NC4 strain [33]. NC4A2 is referred to as "wildtype" hereafter. Cells were grown at 19°C on plastic culture dishes in HL-5 (0.75% proteose peptone, 0.75% thiotone E peptone, 0.5% Oxoid yeast extract, 1% glucose, 2.5 mM Na_2_HPO_4_, and 8.8 mM KH_2_PO_4_, pH 6.5) supplemented with penicillin-streptomycin at 60 U/ml. The *cpnA*^- ^null mutant strain used to test the specificity of the CpnA antisera was created by replacing the *cpnA *gene with the *bsr *gene by homologous recombination in NC4A2 cells.

### Database sequence analysis

The human copine I cDNA sequence was used to search the Dictyostelium cDNA Sequencing Project database at the University of Tsukaba in Japan [15] using BLAST. This search yielded multiple cDNA clones with homologies to human copine I. Two full-length clones, SLI-395 and CFH-205, were obtained and sequenced. The corresponding genes were named, cpnA and cpnB, respectively (GenBank accession numbers AY332759 and AY5993970). The cDNA sequences of cpnA and cpnB were then used to search both the Dictyostelium cDNA and genomic sequence databases [15,34,35]. Using sequences obtained from cDNA and genomic sequence databases, four additional copine genes, cpnC-F, were identified and their corresponding amino acid sequences predicted. Recently, five of the copine genes were identified by the Dictyostelium genome sequence center as predicted genes. The dictybase ID numbers are as follows: cpnA, DDB0215368; cpnB, DDB0216245; cpnC, DDB0216239; cpnD, DDB0216244; cpnE, DDB0216242 [16]. cpnF has not been curated by the sequencing center as a predicted gene. Amino acid sequences were aligned using Lasergene's Megalign Clustal V method.

### CpnA antibody production and purification

Rabbit polyclonal antibodies were raised against a bacterially expressed fragment of CpnA. Sequences encoding the first C2 domain of *cpnA *(CpnA-C2A, amino acids 1–137) were amplified by PCR from the SLI-395 cDNA clone and subcloned into the pGEX-KG plasmid [36]. The pGEX-KG/CpnA-C2A plasmid was transformed into the DH5α strain of *E. coli *for the production of a GST (glutathione S-transferase)-fusion protein. The recombinant GST-CpnA-C2A fusion protein was purified by glutathione agarose affinity chromatography and thrombin cleavage as described in Damer and Creutz [17]. The supernatant containing the CpnA-C2A fragment was further purified by running the solution over a *p*-aminobenzamidine column to remove the thrombin. The concentration of CpnA-C2A in resulting column fractions was determined using the Bio-Rad protein assay. The purified CpnA-C2A fragment was sent to Cocalico Biologicals (Reamstown, PA) for antibody production. The resulting rabbit antisera was tested for CpnA recognition by Western blotting. CpnA antibodies were purified from the antisera using a blotting protocol according to Olmsted [37].

### RT-PCR and Western blot analysis

NC4A2 cells were washed 4 times in PDF starvation buffer (20 mM KCl, 11 mM K_2_HPO_4_, 13.2 mM KH_2_PO_4_, pH 6.4, 1 mM CaCl_2_, 2.5 mM MgSO_2_) and 2.5 × 10^7 ^cells were plated on filters (Millipore, HABPO4700) placed on top of 47 mm Petri dishes with pads (Fisher, 09-753-53C). Dishes were placed in plastic sandwich containers with wet paper towels and allowed to develop at 19°C for various time periods. At 0, 5, 10, 15, 20, and 25 hr, cells were washed off filters by vortexing in PDF buffer and collected by centrifugation. For whole cell protein samples, the pellets were resuspended in sample buffer and boiled for 5 min. For RNA samples, total RNA was isolated from cells by acid guanidinium thiocyanate-phenol-chloroform extraction [37], treated with DNase, and quantified by UV analysis. Whole cell samples from each time point were analyzed by SDS-PAGE at 5 × 10^6 ^cells per lane on a 12% polyacrylamide gel stained with Coomassie Blue. For Western blotting, a 12% polyacrylamide gel with whole cell protein samples was transferred to a polyvinylidene fluoride (PVDF) membrane. The membrane was incubated with blocking buffer (5% dry milk, 0.5% Tween-20 in PBS) for 1 hr at RT, then incubated with purified CpnA antibodies (0.2 μg/ml) in blocking buffer for 1 hour at RT, washed in 0.5%Tween-20 in PBS, and incubated with a peroxidase-conjugated goat anti-rabbit 2° antibody (1:200,000) in blocking buffer for 1 hr at RT. The membrane was washed with 0.5% Tween in PBS and incubated with SuperSignal West Substrate Working Solution (Pierce, Rockford, IL) and exposed to X-ray film.

The ProSTAR™ Ultra HF (High Fidelity) RT-PCR system from Stratagene was used to detect *cpnA *mRNA in total RNA samples from each time point according to manufacturer's instructions. Primers were designed to amplify a 400 bp fragment that crossed an intron. Reactions without reverse transcriptase and with primers to *cinD *mRNA were run as controls.

### Expression of GFP-CpnA

The full length coding sequence of cpnA was amplifed by PCR from the cDNA clone, SLI-395. The PCR fragment was subcloned into a Dictyostelium extrachromosomal plasmid (pTX-GFP, [38]) containing a gene for a variant of green fluorescent protein (GFP, S65A, V68L, and S72A mutations) to produce a fusion protein with GFP at the N-terminus of CpnA (GFP-CpnA). Wildtype Dictyostelium cells were transformed with the plasmid by electroporation and G418 resistant cells were screened for expression of GFP-CpnA by Western blot analysis using antibodies to both CpnA and GFP. As a control, wildtype Dictyostelium cells were also transformed with pTX-GFP without a cpnA cDNA insertion.

### Membrane binding assays

For phospholipid binding assays with CpnA-C2A, bovine brain lipids (Sigma, B-1502, 100 mg) were dissolved in 10 ml of buffer (20 mM HEPES, 30 mM KCl, pH 7.4) and sonicated to form liposomes. Purified CpnA-C2A (8 μg) was incubated for 10 min with 50 μg of the prepared liposomes in the presence of either 2 mM CaCl_2 _or 2 mM EGTA. Liposomes were centrifuged for 10 min in a microfuge at 14,000 RPM. Pellets were resuspended in boiling sample buffer and analyzed on an 8% polyacrylamide SDS gel stained with Coomassie Blue.

For native membrane binding assays of endogenous CpnA and GFP-CpnA, wildtype cells and those expressing GFP-CpnA were disrupted in homogenization buffer (50 mM HEPES, pH 7.4, 150 mM KCl containing either 2 mM CaCl_2 _or 2 mM EGTA) with 2 passes through a French Press. The cell lysates were centrifuged in a Beckman Tabletop centrifuge at 500 × g to pellet unbroken cells and the supernatants were then spun in a Beckman Ultracentifuge at 100,000 × g for 45 min. Supernatants were run over desalting columns in water, lyophilized, and resuspended in sample buffer. The pellets were resuspended in boiling sample buffer. Pellets and supernatants were analyzed by Western Blot using the CpnA antisera and a peroxidase-conjugated goat anti-rabbit secondary antibody (Sigma), or a monoclonal GFP antibody (Sigma) and a peroxidase-conjugated goat anti-mouse secondary antibody (Sigma), and colorimetric detection with 4-chloronapthol.

### Cell fixation and immunofluorescence

Cells expressing GFP-CpnA were allowed to adhere to coverslips for 15 min in a humid chamber, rinsed with PDF buffer, and in most cases overlaid with a thin sheet of agarose [40]. Cells were then fixed with 1% formaldehyde in methanol at -10°C for 5 min, washed three times in PDF buffer, and mounted on slides. To enlarge contractile vacuoles, cells were allowed to adhere to coverslips and incubated in water for 1.5 min before fixation. To label endosomes/lysosomes, cells expressing GFP-CpnA in culture dishes were incubated with 0.02 μm red fluorescent microspheres (Fluospheres, Molecular Probes, F-8786; [41]) diluted 1:100 in HL-5 for 2 hours, washed once, and placed on coverslips for 15 min before fixation. To label phagosomes, cells expressing GFP-CpnA were washed twice in PDF buffer, placed on coverslips for 15 minutes, washed twice with PDF buffer, and incubated with Alexa Fluor-594-labeled yeast (Molecular Probes, Z-23374, 20 mg/ml, then diluted 1:100 in PDF buffer) for 1 hour before fixation. For calmodulin antibody immunofluorescence, fixed cells were rinsed with TBS (Tris-buffered saline) and incubated with a monoclonal antibody to calmodulin (1:400, Sigma, [22]) for 30 min at 37°C. Fixed cells were then rinsed with TBS, incubated with a TRITC-conjugated goat anti-mouse antibody (1:400, Sigma), rinsed with water, and mounted on slides.

### Microscopy imaging of fixed and live cells

For widefield fluorescence imaging, fixed cells were imaged using differential interference contrast and epifluorescence optics on a Nikon TE2000, with a 100× objective, filter cubes for GFP and TRITC, a Cooke Sensicam camera, and Image Pro-Plus software. For confocal imaging, a Nikon laser scanning confocal system equipped with an upright TE800 microscope, a 60× objective, and SimplePCI software, was used. For confocal imaging of nanobeads and labeled yeast, both the argon and He/Ne lasers were used. For imaging of starved cells, cells were washed 3 times in PDF starvation buffer and placed on glass bottom plates. Cells were imaged every 2.5 s using a 60× water dipping objective and the argon laser set at 10% transmittance. For live cell disruption studies, cells were placed on glass bottom dishes in either water or 2 mM EGTA in water. Cells were imaged every 2.5 s using a 60× water dipping objective and the argon laser set at 100% transmittance. Images were cropped, aligned, and adjusted for contrast using Adobe Photoshop Elements 2.0 and/or Microsoft Powerpoint X for Mac. Video sequences and time-lapse montages were made using SimplePCI.

## Authors' contributions

CKD wrote the manuscript, created the figures, and designed and supervised all experiments. CKD also carried out the sequence analysis, microscopy, and image capturing. MB carried out the membrane binding assays. JR carried out the development expression pattern experiments. CIS carried out the fixed cells experiments and assisted with live cell imaging. ESH assisted with the disruption of live cell assays.

## Additional files

All additional files are movies in AVI format except for Movie 9, which is MOV format. Movies 1–8 consist of time-lapse confocal images taken every 2.5 s and are shown 2 images/s so that time is compressed 5×. Movie 9 consists of confocal images that make up a z-series of a single cell.

## Supplementary Material

Additional File 1 A single cell expressing GFP-CpnA that has been starved for 5.5 hrs (same cell shown in Figure 4A). GFP-CpnA translocates from the cytosol to membranes and back to the cytosol once. Click here for file

Additional File 2 A single cell expressing GFP-CpnA that has been starved for 5.5 hrs (same cell shown in Figure 4B). GFP-CpnA translocates from the cytosol to membranes and back to the cytosol twice. Click here for file

Additional File 3 A single cell expressing GFP-CpnA that has been starved for 4 hrs (not shown in Figure 4). GFP-CpnA translocates from the cytosol to membranes and back to the cytosol six times. Click here for file

Additional File 4 Adjacent cells expressing GFP-CpnA that have been starved for 10 hrs. GFP-CpnA translocates from the cytosol to membranes multiple times in each cell. Click here for file

Additional File 5 A small aggregate of cells expressing GFP-CpnA that has been starved for 9.5 hrs (one of these cells is shown in Figure 4C). GFP-CpnA translocates from the cytosol to membranes and back to the cytosol once in four different cells at different times. Click here for file

Additional File 6 Cells expressing GFP-CpnA were placed in water to promote bursting and imaged with a confocal microscope every 2.5 s (same cells as in Figure 5A). Click here for file

Additional File 7 Cells expressing GFP-CpnA were placed in water containing 2 mM EGTA to chelate calcium and imaged with a confocal microscope every 2.5 s (same cells as in Fig. 5B). Click here for file

Additional File 8 Cells expressing GFP alone were placed in water to promote bursting and imaged with a confocal microscope every 2.5 s (same cells as in Fig. 5C). Click here for file

Additional File 9 A z-series of images taken from the bottom to the top of a single fixed cell expressing GFP-CpnA (same cell as in Fig. 6G). Click here for file
